# Editorial: Novel Insights Into Insect Antiviral Immunity

**DOI:** 10.3389/fimmu.2021.740989

**Published:** 2022-01-10

**Authors:** Liang Jiang, Xiao-Qiang Yu, Luc Swevers

**Affiliations:** ^1^ State Key Laboratory of Silkworm Genome Biology, Chongqing Key Laboratory of Sericultural Science, Chongqing Engineering And Technology Research Center for Novel Silk Materials, Southwest University, Chongqing, China; ^2^ Biological Science Research Center, Southwest University, Chongqing, China; ^3^ Guangdong Provincial Key Laboratory of Insect Developmental Biology and Applied Technology, Institute of Insect Science and Technology, School of Life Sciences, South China Normal University, Guangzhou, China; ^4^ Division of Cell Biology and Biophysics, School of Biological and Chemical Sciences, University of Missouri-Kansas City, Kansas City, MO, United States; ^5^ Insect Molecular Genetics and Biotechnology, Institute of Biosciences and Applications, National Centre for Scientific Research Demokritos, Athens, Greece

**Keywords:** insect, virus, infection, immunity, antiviral

Insects are the largest group of animals distributed throughout the earth, including economically important insects (e.g. silkworms, honeybees, pollinators), agricultural and forestry pests (e.g. locusts, stink bugs, armyworms, weevils), virus vectors (e.g. mosquitoes, midges, blackflies), and model organisms (e.g. *Drosophila* in genetics and developmental biology). Viruses are the major pathogens of insects; however, the mechanism of viral infection and antiviral insect immunity is not fully understood. The 15 articles of this Research Topic highlight the latest advances regarding insect antiviral immunity.

Five contributions refer to the interaction between baculovirus and insect host. Jiang et al. reviewed the arms race between silkworm and baculovirus, including the baculovirus invasion mechanism, the silkworm immune response and the viral immune evasion mechanism, and surveyed strategies for the enhancement of host antiviral capacity. The authors also discussed outstanding major issues and future directions of research on silkworm antiviral immunity. Melanization is mediated by the prophenoloxidase (PPO) pathway, which is an important humoral response for killing invading pathogens in insects. Wang et al. identified a conserved PPO activation pathway in *Helicoverpa armigera* and confirmed that the three-step SP41/cSP1/cSP6 cascade can convert PPO into active phenoloxidase (PO), and that the cofactors cSPH11 and cSPH50 can enhance PO activity activated by cSP6. An *in vitro* reconstituted PPO activation cascade can block baculovirus infection, indicating the importance of melanization in controlling baculovirus infection. Baculovirus is characterized by a restricted host range: the silkworm is permissive for BmNPV infection but is a non-permissive host for AcMNPV. Lin et al. found that adenosine signaling was upregulated to enhance host energy levels after infection with non-permissive AcMNPV, and that inhibition of the adenosine receptor (*AdoR*), glycolysis and adenosine transport can decrease ATP content and increase AcMNPV proliferation in BmN cells, suggesting that *AdoR* modulates permissiveness of baculovirus infection *via* regulation of energy metabolism in the silkworm. Viruses also regulate the development and protein modifications of their hosts. Previous studies have shown that newly exuviated fifth instar silkworms infected with BmNPV exhibit delayed maturation. Results from Xu et al. further indicated that day-4 fifth instar larvae infected with BmNPV showed an increase in ecdysone titer and precocious maturation, and RNA-seq was further used to analyze the candidate genes involved in this process. Mao et al. investigated the effect of HSC70-4 deacetylation on BmNPV infection. The authors found that lysine 77 (K77) deacetylation promoted the stability and nuclear import of HSC70-4 and viral proliferation, and that this process may be modulated by the ubiquitin proteasome system.

Some insects serve as vectors to transmit viruses, which adversely affect agricultural production (for plant viruses) and spread human diseases (for arboviruses). Five papers focus on the molecular mechanisms underlying the interactions between viruses and insect vectors. Zhao et al. investigated the proteomic interactions between tomato yellow leaf curl virus (TYLCV) and its whitefly vector and found that the whitefly protein Tid interacted with the coat protein of TYLCV. Tid protein content was increased following viral acquisition, and inhibition of Tid resulted in increased TYLCV replication in whitefly, suggesting the inhibitory role of Tid on viral infection. He et al. found that Toll pathway core genes (*Toll*, *MyD88*, and *Dorsal*) were upregulated in the planthopper vector after infection with rice stripe virus (RSV), and observed direct interactions between the viral nucleocapsid protein and the Toll receptor. RNAi of *Toll* led to increased RSV proliferation and mortality in planthoppers, indicating the antiviral defense of the Toll pathway against the plant virus in the planthopper vector. Many flaviviruses are arboviruses and major human pathogens, including Dengue virus (DENV), Zika virus (ZIKV), West Nile virus, and Yellow Fever virus. Harsh and Eleftherianos summarized recent studies about flavivirus infections and antiviral immune mechanisms and discussed the host tissue homeostasis and pathophysiological defects in mosquitoes and the model insect *Drosophila*. Leite et al. investigated the distinct functional roles of hemocytes at different stages of infection by DENV and ZIKV in mosquitoes. The authors showed that hemocytes were recruited to the midgut in response to virus and that blocking phagocytosis led to decreased viral replication in the midgut. By contrast, phagocytosis by hemocytes was essential to restrict viral dissemination during systemic infection. Results from Weng et al. showed that *TEP1* transcription was induced in mosquitoes following DENV infection, and silencing of *TEP1* resulted in decreased expression of the transcription factor Rel2 and certain antimicrobial peptides (AMPs) as well as increased viral content, suggesting that TEP1 regulates the immune response and consequently limits DENV infection in mosquitoes.

Four other contributions have topics that deal more generally with antiviral pathways and effector molecules. The first topic highlights intracellular and extracellular degradation as crucial for restricting viral infection. Jiang reviewed the main antiviral immune pathways and the virus-modulated signaling pathways in the silkworm; the former includes RNAi and signaling pathways mediated by NF-κB, Imd, STING and JAK/STAT while the latter includes the PPO, PI3K/Akt, and ERK pathways. Targeting these virus-modulated pathways by gene editing or inhibitors can enhance host antiviral capacity. Feng et al. reviewed the roles of (both validated and potential) AMPs in insect antiviral immune response and their possible mechanisms of synthesis and action. A second topic emphasizes the requirement for intercellular communication to mount systemic immune responses. Wang summarized the intercellular communications in insect antiviral immunity, including protein-based and virus-derived RNA-based cell-cell communications, and focusing on the signaling pathway that induces the production of potential cytokines. Another article focuses on the symbiont *Wolbachia*, a maternally transmitted bacterium in insects, which was recently discovered to protect insects against RNA viruses. Pimentel et al. described the main advances and possible mechanisms of the antiviral effect of *Wolbachia*. The authors also discussed the potential antiviral effect of *Wolbachia* in wild insect populations and its ecological relevance.

A final article presented by Lin et al. also adds a piece of interesting data on the regulation of host genes by virus-encoded miRNAs. The authors showed that the expression levels of BmCPV-miR-1 and BmCPV-miR-3 were increased while their common target host gene *BmRan* was inhibited in silkworms infected with cypovirus. It is proposed that the two miRNAs can inhibit *BmRan* expression and promote viral proliferation.

In summary, all published articles describe exciting new data of insect immunity against viral infection and provide new mechanisms of resistance and targets for pest control that can also have relevance for antiviral research in humans.

## Author Contributions

All authors listed have made a substantial, direct and intellectual contribution to the work, and approved it for publication.

## Funding

This work was funded by the National Natural Science Foundation of China (No. 32170524 and No. 31970474) and the Natural Science Foundation of Chongqing, China (cstc2020jcyj-cxttX0001). Luc Swevers acknowledges support of this work by the project ‘An Open-Access Research Infrastructure of Chemical Biology and Target-Based Screening Technologies for Human and Animal Health, Agriculture and the Environment (OPENSCREEN-GR)’ (MIS 5002691) which is implemented under the Action ‘Reinforcement of the Research and Innovation Infrastructure’, funded by the Operational Programme ‘Competitiveness, Entrepreneurship and Innovation’ (NSRF 2014-2020) and co-financed by Greece and the European Union (European Regional Development Fund).

## Conflict of Interest

The authors declare that the research was conducted in the absence of any commercial or financial relationships that could be construed as a potential conflict of interest.

## Publisher’s Note

All claims expressed in this article are solely those of the authors and do not necessarily represent those of their affiliated organizations, or those of the publisher, the editors and the reviewers. Any product that may be evaluated in this article, or claim that may be made by its manufacturer, is not guaranteed or endorsed by the publisher.

